# Prevalence and drug resistance profile of *Mycobacterium tuberculosis* isolated from pulmonary tuberculosis patients attending two public hospitals in East Gojjam zone, northwest Ethiopia

**DOI:** 10.1186/s12889-015-1933-9

**Published:** 2015-06-20

**Authors:** Kelemework Adane, Gobena Ameni, Shiferaw Bekele, Markos Abebe, Abraham Aseffa

**Affiliations:** Institute of Biomedical Sciences, College of Health Sciences, Mekelle University, PO Box 1872, Mekelle, Ethiopia; Aklilu Lemma Institute of Pathobiology, Addis Ababa University, PO Box 1176, Addis Ababa, Ethiopia; Armauer Hansen Research Institute, PO Box 1005, Addis Ababa, Ethiopia

**Keywords:** *Mycobacterium tuberculosis*, Drug resistance, MDR-TB, Ethiopia

## Abstract

**Background:**

The spread of multidrug-resistant tuberculosis (MDR-TB) strains has become a challenge to the global TB control and prevention program. In Ethiopia, particularly in rural areas, information on drug-resistant TB is very limited. In this study, we determined the drug resistance patterns of *Mycobacterium tuberculosis* (*M. tuberculosis*) isolates from pulmonary TB patients attending two public hospitals in the East Gojjam zone of northwest Ethiopia.

**Methods:**

A cross-sectional study was conducted between May 2011 and January 2012 using Region of difference-9 (RD9) typing for the identification of species mycobacterium. Drug susceptibility testing (DST) of *M. tuberculosis* isolates to the first-line drugs: isoniazid, rifampicin, ethambutol and streptomycin was performed by the indirect proportion method on Middle brook 7H10 Agar media.

**Results:**

Out of 385 pulmonary TB suspects studied, 124 (32.2 %) were culture positive among which 120 were *M. tuberculosis* strains. Susceptibility testing was performed for 89 isolates. Resistance to at least one drug was 15.58 % ([12/77], 95 % CI: 7.48-23.68) among newly diagnosed and 50.0 % ([6/12], 95 % CI: 21.71-78.29) among previously treated cases. Resistance among newly diagnosed patients was most common for streptomycin 5.19 % (4/77) and ethambutol 5.19 % (4/77) followed by rifampicin 3.89 % (3/77). Among retreatment cases, isoniazid resistance was most frequent in which 33.33 % (4/12) of the isolates were resistant. MDR prevalence was 1.29 % (1/77) for newly diagnosed and 16.67 % (2/12) for retreatment cases. In a multivariate logistic regression analysis, age group of 25–34 years (adjusted OR = 4.24; 95 % CI: 1.02-17.5; P = 0.046) and previous history of treatment (adjusted OR = 5.42; 95 % CI: 1.56-27.49; P = 0.01) were independently associated with anti-TB drug resistance.

**Conclusions:**

In general, the magnitude of anti-TB drug resistance including MDR-TB was comparable to previous studies in other areas of Ethiopia. However, rifampicin resistance was high, which could suggest the potential for a rise in the incidence of MDR. Therefore, re-enforcing TB control programs should be considered by the concerned public health authorities.

## Background

The emergence of drug-resistant tuberculosis is a critical threat to tuberculosis (TB) control and is a major public health concern in several countries. Multidrug-resistant TB (MDR-TB), defined as resistant to at least isoniazid and rifampicin, is emerging as a major clinical and public health challenge in areas of sub-Saharan Africa [1]. Ethiopia is one of the high TB and HIV burdened countries in sub-Saharan Africa which ranks 7th among the 22 high TB burden countries in the world [1]. According to the WHO 2013 report, the prevalence and incidence rates of TB in the country were 224/100,000 and 247/100,000 populations, respectively. The rate of MDR-TB was estimated to be 1.6 % and 12 % of new and retreatment cases, respectively [1].

In countries with high burden of TB, continuous surveillance and regular monitoring of drug resistance based on routine drug susceptibility testing (DST) of TB patients is essential to assess the magnitude and trends of anti-TB drug resistance [1, 2]. However, in Ethiopia, there is limited capacity to perform culture and DST of *M. tuberculosis,* even from patients suspected of harbouring drug-resistant strains. As a consequence, many of MDR-TB patients remain undiagnosed which negatively impacts the global strategy of halting and eliminating TB [3].

Few studies previously conducted in Ethiopia indicated drug resistance proportions ranging from 14.6 %-30.1 % [4–6] and 11.1 %-42.1 % [4, 5] among new and previously treated patients, respectively. However, most studies were conducted in urban areas and might not be representative for rural remote regions of the country. Besides, in Ethiopia, the socioeconomic, lifestyle and environmental conditions differ across regions, varying from agricultural based to pastoral communities and from semi-arid to humid fertile areas [7], and could affect the distribution of drug-resistant *M. tuberculosis* strains [8]. The current study was conducted among patients attending health facilities in the East Gojjam zone of the Amhara region in northwest Ethiopia. East Gojjam zone is one of the rural areas where the communities lead their life mainly by farming and to date there is no report on anti-tuberculosis drug resistance from this part of the country. This study therefore determined the prevalence of drug-resistant and MDR-TB among pulmonary TB patients attending two public hospitals in the region from May 2011 to January 2012.

## Methods

### Study setting

This cross-sectional study was conducted in Debre Markos Referral and Mota District hospitals located in the East Gojjam zone of the Amhara region, northwest Ethiopia. The prevalence of all forms of TB and smear-positive TB in the Amhara Regional State was reported to be 643 and 168 per 100,000 populations, respectively [9]. East Gojjam zone is divided into 18 administrative districts and agriculture is the major source of income for more than 90 % of its population. There are two public hospitals, more than 18 health centers, about 384 health posts, and 5 higher and 25 medium private clinics providing service for about 3.5 million people in their catchment areas. In addition to the two hospitals, all the 18 health centers provide sputum smear microscopy and directly observed treatment short course (DOTS) service. Moreover, the 5 higher and most medium private clinics have currently implemented the DOTS service [10, 11]. We conducted the current study at the two public hospitals due to the fact that they represent the largest center for TB diagnosis (referral sites for rural communities) and considering feasibility.

### Study population

From May 2011 to January 2012, a total of 385 pulmonary TB suspected patients presenting at the two hospitals were consecutively enrolled. Both smear-positive and smear-negative patients were included. Inclusion criteria were: age ≥ 15 years, willingness to participate in the study (with informed consent including for HIV testing) and clinical suspicion of pulmonary TB strong enough to warrant a direct sputum smear for acid-fast bacilli (AFB). Prior to sputum collection, data on demographic information and history of previous treatment were collected using a structured questionnaire. Then, three consecutive sputum samples (spot, morning and spot) were collected from each patient. Direct microscopic examination of sputum for AFB after Ziehl- Neelsen staining was performed at the laboratories of the respective hospitals. Then samples were stored at 4 °C for a maximum of 5 days until transported in a cold box to the Armauer Hansen Research Institute (AHRI) in Addis Ababa for culturing.

### HIV testing

Testing for HIV was done according to the current National algorithm recommended by the Federal Ministry of Health of Ethiopia. Two rapid HIV tests, HIV (1 + 2) Rapid Test Strip (KHB) and Stat-Pak were run sequentially. Samples were tested first with KHB. Positive samples were confirmed with Stat-Pak. Discordant results were resolved using a third confirmatory test kit, HIV-1/2 Unigold Recombinant assay. Pre and post-test HIV counseling was provided for all consenting individuals.

### Mycobacterial Culture

Sputum specimens were processed by the Petroff’s method following the standard operational procedures. In brief, an equal volume of sputum specimen was mixed with an equal volume of 4 % NaOH and concentrated at 3000 rpm for 15 min. The sediment was neutralized with 2 N HCL, using phenol red as indicator, to prepare the inoculums for culture. Then, three Lowenstein-Jensen slants, two containing 0.75 % glycerol and one containing 0.6 % pyruvate, were inoculated per each sputum sample. Culture tubes were incubated at 37 °C and inspected for growth of colonies weekly for up to 8 weeks. Mycobacteria were confirmed with acid-fast bacilli (AFB) staining.

### Region of difference 9 (RD9) deletion typing for identification of *M. tuberculosis*

DNA was extracted by heat killing of the mycobacterial colonies. Briefly, heat killed bacterial suspensions were obtained by heating cultures suspended in Tris-EDTA (TE) at 80 °C for one hour followed by incubation in an ultrasonic bath for 5 min. Region of Difference analysis was performed by using RD9 FlankFW, RD9 Int and RDFlankRev primers as described previously [12]. Interpretation of results was made on the basis of detection of bands of different sizes [13]. For quality control, *M. tuberculosis* H37Rv and *M. bovis* BCG (as positive control) and water (as a non-template control) were included.

### Conventional drug susceptibility testing

Drug susceptibility testing (DST) was performed by the indirect proportion method on the enriched Middlebrook 7H10 agar media using a 24 well plate as recommended by Bert van Klingeren et al. [14]. Stock solutions of each drug were prepared using the appropriate solvent and the following final drug concentrations (critical concentrations) were used: isoniazid (INH), 0.2 μg/ml, rifampicin (RIF),1.0 μg/ml, ethambutol (EMB), 5.0 μg/ml and streptomycin (STM), 2.0 μg/ml as recommended elsewhere [15]. Standardized bacterial suspension for each isolate was distributed in 10 μl volumes into 9 wells of the 24-well plate containing 7H10 medium with a precise concentration of the selected anti-TB drugs and into the 10th well containing drug free medium. The 11th well of each plate was inoculated with a mycobacterial solution containing 1 % of the suspension (1:100 suspensions). This was followed by incubation at 35 °C in the presence of sufficient humidity and bacterial growth was checked on the 12th, 19th and 28th days. If the growth on drug containing media with 1:1 bacterial suspensions was greater than the growth on drug free control with 1:100 bacterial suspensions, the isolate was defined as resistant and if it was lower, the isolate was defined as susceptible to that particular drug. The test was repeated if the growth was equal or nearly equal for both. For quality control, the reference strain of *M. tuberculosis* H37Rv (ATCC 27294), which is susceptible to the entire standard anti tuberculosis drugs, was used in each batch of media prepared with drugs.

Resistance to a particular drug with or without resistance to any of the other anti-TB drugs was denoted as ‘any resistance’. An MDR-TB patient was defined as one whose sputum isolates showed resistance to isoniazid and rifampicin with or without resistance to other anti-TB drugs. Poly-resistance was defined as resistance to more than one drug with the exception of the combination of isoniazid and rifampicin.

### Data analysis

Data were entered using Epi Data entry version 3.1 software and analyzed using SPSS version 21. Differences between sputum culture positive and culture negative groups were investigated using the *x*^2^ test (categorical variables). Age was categorized into four levels. Bivariate and multivariate logistic regression analysis was performed to examine the association of independent variables with resistance outcome. Covariates with p-values of ≤ 0.25 in the bivariate analysis were included in the multivariate model. HIV status was considered as a potential confounder and hence was assessed. Accordingly, the multivariate model consisted of age (age group of 15–24 years as a referent), treatment category (new cases as referent) and HIV status (HIV-negative patients as a referent). Comparisons between subgroups (new cases vs. retreatment cases, HIV-positive vs. HIV-negative, and age groups) with resistance outcome were expressed as odds ratios (OR) with a 95 % confidence interval (CI). P-values of ≤ 0.05 were considered as statistically significant.

### Ethical issues

Ethical clearance was obtained from Aklilu Lemma Institute of Pathobiology, Addis Ababa University (AAU) and AHRI/ALERT research ethics review committees. Written informed consent was obtained from the study participants and assent from parents/guardians for those under 18 years of age. For illiterate participants, data collectors informed each respondent and confirmed the willingness of participants by signing on the informed consent sheet. Moreover, confidentiality was assured for all information provided. Confirmed MDR cases were reported to the study hospitals for treatment.

## Results

### Characteristics of the study population

Of 385 pulmonary TB (PTB) suspects enrolled, 244 (63.4 %) were males and 141 (36.6 %) females with an age range of 15 to 73 and mean age of 36.8 years. The majority of the study participants, 326 (84.7 %), were rural residents and more than three-fourths, 295 (76.6 %) were farmers. Illiterates numbered 267 (69.4 %) and 118 (30.6 %) had completed primary school or above. Ninety-two (23.9 %) of the study participants reported close contact with a TB patient and mostly (89.0 %) at home. Eighty-nine (23.1 %) were HIV positive, 79 (20.5 %) were smear-positive, and 68 (17.7 %) had a previous history of anti-TB therapy.

### Culture result and relation to patient characteristics

Of 385 sputum samples, 124 (32.2 %) (from 82 male and 42 female patients) were culture positive. Eight (2.1 %) were contaminated. Seventy-seven (97.5 %) of the smear-positive and 47 (18.1 %) of the smear-negative sputum samples were culture positive. Two (2.6 %) smear-positive specimens were culture negative. Thirty-four (36.9 %) of the 92 participants who reported close contact with a TB patient were culture positive. Positive cultures were more frequent among HIV-negative than HIV-positive participants, but the difference was not statistically significant (P = 0.093). There was no significant difference between the culture-positive and the culture-negative groups in terms of HIV status, age distribution, sex, and history of previous TB treatment (Table 1).

**Table 1:** Baseline characteristics of participants enrolled in the study and their association with culture outcome as analyzed by the x2 test

Characteristic	Participants with positive sputum culture (n = 124), n (%)	Participants with negative sputum culture (n = 261), n (%)	*P* value^*^
Sex			
Male	82 (66.1)	162 (62.0)	0.256
Female	42 (33.9)	99 (38.0)	
HIV status			
HIV-negative	102 (82.3)	67 (25.7)	0.093
HIV-positive	22 (17.7)	194 (74.3)	
Previous history of treatment			
No	107 (86.3)	51 (19.5)	0.198
Yes	17 (13.7)	210 (80.5)	
Sputum microscopy			
Smear-positive	77 (62.1)	2 (0.7)	< 0.001
Smear-negative	47 (37.7)	259 (99.3)	

^*^Comparing participants with a positive culture vs. participants with a negative culture. TB, Tuberculosis; HIV, Human Immunodeficiency Virus

### Region of difference (RD) based species typing

Region of difference based multiplex PCR for RD9 (RD9 analysis) showed that out of 124 isolates, 120 were *M. tuberculosis* species. The remaining 4 isolates failed to show any band on RD9 typing. No *M. bovis* was identified in this study.

### Drug susceptibility patterns

Phenotypic drug susceptibility testing results were available for 89 (74 %) of the 120 isolates. However, the socio-demographic and clinical characteristics of patients whose isolates were not available for drug susceptibility testing were not significantly different from those included except that the loss was relatively higher in smear-negative culture positive cases.

Resistance to at least one drug was detected in 18 (20.23 %, 95 % CI: 11.86-28.54) of the isolates of which 12 (20.7 %) were from males and 6 (19.4 %) from females. The highest proportions of any drug resistance were observed in STM 7 (7.87 %, 95 % CI: 2.3-13.5) followed by INH 6 (6.74 %, 95 % CI: 1.51-11.89) and EMB 6 (6.74 %, 95 % CI: 1.51-11.89). Three isolates (3.37 %, 95 % CI: 0.0-7.17) were MDR and 2 (2.24 %) were poly resistant. One MDR isolate was resistant to all the four anti-TB drugs tested. A summary of drug susceptibility pattern of the isolates is shown in Table 2.

**Table 2:** Drug resistance patterns of *M.tuberculosis* strains isolated from newly diagnosed and previously treated pulmonary TB patients attending two public hospitals in East Gojjam zone, northwest Ethiopia

Drug resistance pattern	Isolates from new cases, n (%)	Isolates from previously treated cases, n (%)	Total, n (%)	95 % CI
	77 (86.5)	12 (13.5)	89 (100.0)	
Any resistance				
Resistance to any drug	12 (15.58)	6 (50.0)	18 (20.23)	11.86-28.54
Any INH	2 (2.59)	4 (33.33)	6 (6.74)	1.51-11.89
Any RIF	3 (3.89)	2 (16.67)	5 (5.62)	0.82-10.38
Any EMB	4 (5.19)	2 (16.67)	6 (6.74)	1.51-11.89
Any STM	4 (5.19)	3 (25.0)	7 (7.87)	2.3-13.52
Mono resistance				
INH alone	0	1 (8.3)	1 (1.12)	0.0-3.27
RIF alone	2 (2.59)	0	2 (2.24)	0.0-5.25
EMB alone	4 (5.19)	1 (8.33)	5 (5.62)	0.82-10.38
STM alone	3 (3.89)	0	3 (3.37)	0.0-7.14
Resistance to more than one drug				
INH + RIF only (MDR)	1 (1.29)	1 (8.33)	2 (2.24)	0.0-5.25
INH + RIF + EMB + STM only	0	1 (8.33)	1 (1.12)	0.0-3.27
INH + STM only	0	2 (16.67)	2 (2.24)	0.0-5.25

CI, confidence interval; TB, tuberculosis; INH, isoniazid; STM, streptomycin; RIF, rifampicin; EMB, ethambutol

Anti-TB drug resistance was more frequent among retreatment patients than newly diagnosed cases (50.0 % vs.15.58 %) and this difference was statistically significant (crude OR = 5.42; 95 % CI: 1.49-19.64, P = 0.01). The isolate which was resistant to the four anti-TB drugs tested was also isolated from a previously treated patient.

The HIV prevalence among the study population for whom drug resistance testing result available (n = 89) was 19.1 % (17/89). The occurrence of drug resistance was not related to HIV infection status (crude OR = 2.72; 95 % CI: 0.85- 8.88; P = 0.093), to positivity by microscopy of sputum smears (crude OR = 1.08; 95 % CI: 0.36-3.24; P = 0.88) or gender (crude OR = 1.52; 95 % CI: 0.48-4.69; P = 0.48). However, we found a significant association between age and anti-TB drug resistance; those in the group of 25–34 years were more likely to have drug-resistant TB (crude OR = 4.67; 95 % CI: 1.25-17.44; P = 0.022) as compared to patients in other age groups. All the three MDR strains were also isolated from rural HIV-seronegative patients in the age group of 25–34. After fitting into the multivariate logistic regression model, age (adjusted OR = 4.24; 95 % CI: 1.02-17.5; P = 0.046) and previous history of treatment (adjusted OR = 5.42; 95 % CI: 1.56-27.49; P = 0.01) remained independently associated with occurrence of anti-TB drug resistance (Table 3).

**Table 3:** Bivariate and multivariate analysis for patient related risk factors for any drug resistance among pulmonary TB patients attending two public hospitals in East Gojjam zone, northwest Ethiopia (n = 89)

Characteristic	Any drug resistance		COR (95 % CI)	P-value	AOR (95 % CI)	*p*-value
	Yes, n (%)	No, n (%)				
Sex						
Male	13 (72.2)	45 (63.4)	1.52 (0.48-4.69)	0.48		
Female	5 (27.8)	26 (36.6)				
HIV-status						
HIV-positive	6 (33.3)	11 (15.5)	2.72 (0.85-8.81)	0.093	2.76 (0.72-10.54)	0.14
HIV-negative	12 (66.7)	60 (84.5)				
Age group (in years)						
15-24	4 (22.2)	28 (39.4)				
25-34	10 (55.6)	15 (21.1)	4.67 (1.25-17.44)	0.022	4.24 (1.02-17.5)	0.046
35-44	3 (16.6)	17 (23.9)	1.24 (0.25-6.21)	0.79	0.99 (0.18-5.65)	0.99
≥45	1 (5.6)	11 (15.6)	0.64 (0.064-6.35)	0.71	0.59 (0.05-6.36)	0.66
Previous history of treatment						
Yes	6 (33.3)	6 (8.4)	5.42 (1.49-19.64)	0.01	6.55 (1.56-27.49)	0.01
No	12 (66.7)	65 (91.6)				
Smear microscopy						
Smear-positive	12 (66.7)	46 (64.8)	1.087 (0.36-3.24)	0.88		
Smear-negative	6 (33.3)	25 (35.2)				

AOR, Adjusted Odds Ratio; HIV, Human Immunodeficiency Virus; CI, Confidence Interval, COR, Crude Odds Ratio

## Discussion

In this study, about a quarter of *M.tuberculosis* isolates were resistant to at least one of the anti-TB drugs tested (20.23 %). This is higher than a previous report in Gondar (6.7 %) [16] but comparable to a resistance proportion reported in Addis Ababa (22.3 %) [4]. However, a relatively higher resistance proportion (60.8 %) has been reported in another study from Addis Ababa [17]. The possible explanation for this difference could be due to the fact that this study was conducted at St Peter’s TB specialized hospital in Addis Ababa which is a referral site for complicated TB cases from all parts of the country. When compared to studies from other African countries, our figure is also higher than a report from South Africa (7.3 %) [18], but lower than a Kenyan study report (30.1 %) [19].

The most frequent resistance proportion was observed in STM (7.87 %). This drug has been in use since the beginning of TB chemotherapy and hence a higher proportion of STM resistance is expected. However, our figure is lower than previous studies in Ethiopia which reported 26.6 % [20] and 19.0 % [4] of STM resistance. The relatively lower STM resistance in our study could be attributed to time differences in that STM is no more a first line drug (for most cases at least), since the intensive phase was replaced with oral drugs which could lead improved proportions of sensitive isolates with time in recent years. The decreasing proportion of STM resistance could be an advantage as the drug is in the regimen for the treatment of TB patients in category II and for other bacterial infections.

Resistance to INH in our study was 6.74 % which is slightly higher than an earlier report in Addis Ababa (5.5 %) [20] but lower than other reports in Ethiopia in which 17 % [4] and 29.4 % [21] of the isolates were INH resistant. Compared to recent studies in other regions of Africa, our figure is comparable to a report in Rwanda (6.2 %) [22], higher than from Uganda (3.2 %) [22, 23], but lower than a finding in Kenya (12.9 %) [19]. Previous studies conducted in 2002 and 2006 in Addis Ababa reported that 4.1 % [5] and 2.3 % [17] of the isolates were INH mono resistant in new cases and zero in previously treated cases [5, 17]. In our study, INH mono-resistance was detected only in 1 (1.1 %) of the isolates from a previously treated case.

The proportion of EMB resistance in this study (6.74 %) is higher than previous findings in Ethiopia which ranged from 0.0 % to 5.2 % [4, 24, 25]. Monoresistance to EMB was also higher in new cases (5.19 %). This implies the existence of ongoing transmission of drug-resistant strains in the study sites and could indicate weakness in TB prevention and control measures as it is accompanied by mono-resistance to RIF in new cases as well. Since EMB enhances the effect of many other drugs including beta-lactam drugs on different mycobacterial species, the rise of EMB resistance affects the advantage that could be exploited to develop a regimen for the management of MDR-TB [26].

Although the lowest proportion of resistance observed in this study was in RIF (5.62 %), this is higher than previous reports in Ethiopia (0.1 % to 1.4 %) [5, 24, 25]. Monoresistance to RIF was observed in 2.59 % of the isolates which is also higher than previous reports [5, 24]. The relatively higher proportion of resistance to this drug in this study might be attributed to the fact that RIF is largely used in Ethiopia for the treatment of other bacterial infections. Since RIF is the most important drug to treat TB, emergence of resistance to this drug has huge implications for TB control policies. Even if the small sample size might limit us to generalize, our findings suggest that the precursors of MDR-TB are increasing in the study areas as RIF resistance predicts MDR [26]. This could hamper the effectiveness of the new RIF based six-month treatment regimen adopted by the Federal Ministry of Health of Ethiopia. Hence, proper use of this drug should be ensured in the study sites.

In this study, two factors were found to be associated with anti-TB drug resistance; age group of 25–34 years and having had a previous history of TB treatment. Several studies documented the association between a previous history of TB treatment and anti-TB drug resistance [4, 25, 26]. With regard to age group, it is difficult to directly compare with previous reports as different studies used different cut-off points for age groups. A study in South Africa, for instance, showed that age groups of 35–54 and > 54 years were strongly associated with anti-TB drug resistance [27]. The association observed in our study cloud be due to the high-risk behavior of young people and hence a tendency to interrupt TB treatment.

Multi drug resistance (MDR) was observed in 3 (3.37 %) isolates in which 2 (16.67 %) were from previously treated patients and one (1.29 %) from a newly diagnosed case. Previous studies reported MDR proportions ranging from 0.6 % to 2.7 % [4, 17, 24, 28] and 3.5 % to 15.7 % [5, 21, 29] among new and previously treated patients, respectively. Our result of MDR-TB among newly diagnosed patients is higher than a report in Addis Ababa (0.6 %), a study in major towns of Amhara region (1.0 %) [24] and the WHO estimate of 0.1 % primary MDR-TB in Ethiopia [1]. This indicates that there is a progressive increase of MDR over years imposing a serious public health threat. However, it is also worthy to mention that the current magnitude of anti-TB drug resistance in the study sites might have changed since the isolates were collected nearly four years ago. We recommend further large scale studies to be conducted in the underlying population to quantify the problem more precisely.

Although few studies have reported an association between HIV infection and development of drug resistance [30], our study did not find any association between HIV infection status and anti-TB drug resistance. This lack of association between HIV infection status and mycobacterial drug resistance is consistent with the results of earlier studies in Addis Ababa [4, 5] and studies from other sub-Saharan African countries [31, 32]. In our study, the lack of association could be due to the low number of known HIV positive cases included in the study.

Our study was not without pitfalls. First, as the study was conducted among TB patients seeking treatment only at the two hospitals in the zone (health centers and private clinics not included), data may not be representative of TB patients in the population of East Gojjam zone. Moreover, the fact that our sample size was small might have influenced our estimation of anti-TB drug resistance proportion in the study sites. However, the current study provides important data regarding drug resistance in rural hospital settings and could be used by authorities in the region to initiate appropriate interventions.

## Conclusions

Overall, anti-TB drug resistance including MDR-TB is comparable to previous studies in other parts of Ethiopian. However, the relatively higher rate of RIF resistance observed in our study signals the danger of increasing MDR-TB in the study areas in the future. On the other hand, the higher level of MDR-TB among previously treated patients suggests the need to strengthen the DOTS strategy and the capacity of laboratories to undertake DST especially among previously treated patients.

## Abbreviations

AHRI/ALERT: Armauer Hansen Research Institute/All African Leprosy, Tuberculosis and Rehabilitation Training Center
DOTS: Directly observed treatment short course
DST: Drug susceptibility testing
EMB: Ethambutol
HCL: Hydrochoric acid
HIV: Human Immunodeficiency Virus
INH: Isoniazid
NaOH: Sodium hydroxide
MDR: Multidrug-resistance
OR: Odds ratio
RD: Region of difference
RIF: Rifampicin
STM: Streptomycin
TB: Tuberculosis
WHO: World Health Organization
